# The effectiveness of a knowledge translation intervention on the implementation of NEWS2 in nursing homes, a pragmatic cluster RCT

**DOI:** 10.1186/s13012-024-01392-6

**Published:** 2024-09-11

**Authors:** Birgitte Graverholt, Birgitte Espehaug, Donna Ciliska, Thomas Potrebny

**Affiliations:** 1 Department of Health and Caring Sciences, Faculty of Health and Social Sciences, Bergen, Norway; 2 Department of Health and Functioning, Faculty of Health and Social Sciences, Western Norway University of Applied Sciences, Bergen, Norway; 3 Faculty of Health Sciences, McMaster University, Toronto, Canada

**Keywords:** Integrated knowledge translation, KT, Implementation, Trial, Cluster-RCT, NEWS2, Older adults, Nursing home, Long-term care

## Abstract

**Background:**

Improving the uptake of relevant and reliable research is an important priority in long-term care to achieve sustainable and high-quality services for the increasingly older population.

**Aim:**

The purpose was to assess the effectiveness of a tailored, adaptive and a multifaceted KT capacity program, relative to usual practice, on the implementation of National Early Warning Score 2 (NEWS2).

**Methods:**

This study was carried out as a pragmatic cluster-randomized controlled trial. The capacity program consisted of an educational part to address implementation capacity gaps and a facilitation-upon-implementation part to address a relevant knowledge gap in nursing homes. A collective decision was made to address the challenge of early detection of clinical deterioration among nursing home residents, by implementing the (NEWS2) as clinical innovation.

Public nursing homes in a Norwegian municipality (*n* = 21) with a total of 1 466 beds were eligible for inclusion. The study-period spanned over a 22-month period, including a 12-month follow-up.

Data was extracted from the Electronic Patient Journal system and analyzed using multilevel growth model analysis.

**Results:**

The intervention had a large effect on the use of NEWS2 among care staff in intervention nursing homes, compared to the control group (standardized mean difference, d = 2.42). During the final month of the implementation period, residents in the intervention group was assessed with NEWS2 1.44 times (95% CI: 1.23, 1.64) per month, which is almost four times more often than in the control group (mean = 0.38, 95% CI: 0.19, 0.57). During the follow-up period, the effect of the intervention was not only sustained in the intervention group but there was a substantial increase in the use of NEWS2 in both the intervention (mean = 1.75, 95% CI: 1.55, 1.96) and control groups (mean = 1.45, 95% CI: 1.27, 1.65).

**Conclusions:**

This tailored implementation strategy had a large effect on the use of NEWS2 among care staff, demonstrating that integrated knowledge translation strategies can be a promising strategy to achieve evidence-based care in the nursing home sector.

**Trial registration:**

ISRCTN12437773. Registered 19/3 2020, retrospectively.

**Supplementary Information:**

The online version contains supplementary material available at 10.1186/s13012-024-01392-6.

Contributions to the literature

- The IMPAKT study aimed to improve knowledge translation (KT) capacity in nursing homes, using integrated knowledge translation (IKT) as a foundation.
- A tailored, adaptive and a multifaceted KT capacity program had a large effect, relative to usual practice, on the implementation of National Early Warning Score.
- Our study shows that a genuine IKT partnership with high levels of commitment between researchers and a nursing home organization can improve KT capacity and effectiveness of clinical innovations.

## Background

While gains in life expectancy are celebrated, they pose significant challenges to healthcare systems’ long-term care (LTC). The need for LTC is highest among people 80 years and older, a segment expected to double in many countries by 2050 [1]. Concurrent with the increasing demand for LTC is the need to prioritize sustainable and evidence-based care. Key to meeting this complex, yet well-known challenge, is to develop and maintain effective knowledge translation (KT) strategies [2–4].

KT is broadly explained as activities and processes aimed at improving the uptake of relevant and reliable research to improve care [5]. Essentially, KT as a concept and process confronts the multilayered challenge of getting reliable research results into routine healthcare practice [6, 7]. An exponential growth in KT literature and implementation science in recent years, indicates a shift towards dealing with the knowledge-to-action challenges [8]. LTC appears to lag behind in this field [4, 9] and the WHO has highlighted the know-do gap in healthcare settings for the elderly as a key challenge that urgently needs to be addressed [10]. Nevertheless, implementation strategies are too often designed unsystematically and fail to address key contextual components that influence uptake of research into practice [11].

**Fig. 1:**
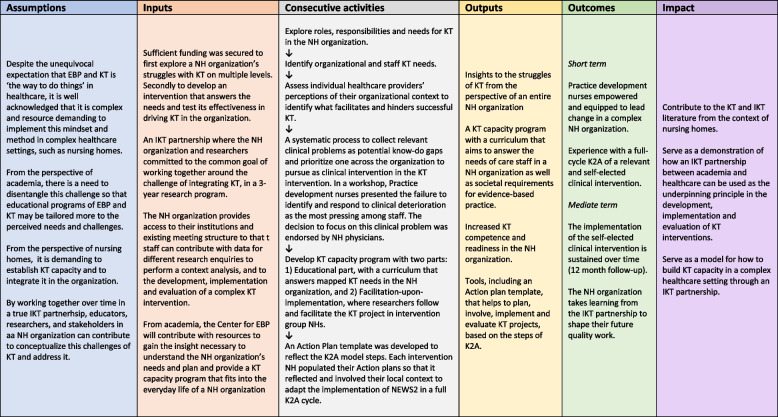
Logic model of the IMPAKT in nursing homes study

Integrated knowledge translation (IKT) has emerged as a participatory research strategy aimed at improving the relevance of research and the uptake of research findings into routine care [12, 13]. IKT partnerships are formed by researchers, knowledge users and decision makers who engage in a reciprocally relevant research project, as equal partners in the research process including setting the research goals [14]. In the case of our study, an IKT partnership was formed upon writing the proposal to a research funding call with the goal to explore and improve the integration of KT into nursing home practice [15]. This complex challenge fits the idea of IKT partnerships, that complex problems require a solution with involvement by multiple levels of perspectives [13, 16] underlying assumptions of this study is presented in a Logic model (Fig. 1).

Several systematic reviews suggest that multifaceted and tailored implementation strategies can be effective and lead to better outcomes compared to single component strategies on clinical practice outcomes [9, 17, 18]. This fits well with the core of IKT principles, where knowledge users collaborate with researchers across all aspects of the research to meet the multilayered needs in the knowledge use context [14, 19].

Early warning scores (EWS) were developed to support healthcare professionals to identify and respond to patients who are at risk of deterioration by assessing vital signs [20]. A scoping review that mapped the use of EWS among older adults outside of hospital settings found that there was little research on its use and implementation [21]. Despite this scarcity of scientific evaluations the use of EWS in is both recommended and used outside hospital [20–24].

In our IKT partnership, stakeholders jointly decided to address early detection of deterioration of acute illness among nursing home residents, using the National Early Warning Score 2 (NEWS2) [20, 22]. NEWS2 is a validated tool originally developed for acute care settings, but its use has expanded outside hospital settings [21, 23, 25].

The overall aim of the IMPAKT study was to improve KT capacity in a nursing home organization. For this trial, the purpose was to assess the effectiveness of a tailored, adaptive and a multifaceted KT capacity program, relative to usual practice, on the implementation of NEWS2.

## Methods

This study is part of the larger IMPAKT in nursing homes study, an IKT study [26] (Fig. 1).

### Trial design

The study was conducted as a pragmatic two-arm cluster randomized controlled trial (RCT).

### Setting and participants

The responsibility for LTC in Norway is placed at the municipal governmental and geographical level. Around 10% of beds in nursing homes are used for short-term purposes, such as post-acute care and rehabilitation, leaving the greater part for long-term beds. Both types of beds offer 24/7 services on a needs-based admission, and when it is not possible to provide the level of care needed by home-based nursing.

This study took place in a Norwegian urban-suburban municipality serving a general population of 292 482 [27]. Twenty-one public nursing home facilities in the municipality were administered in one nursing home organization and all were eligible for inclusion. In total, these nursing homes had 1466 beds, and the size of the NH facilities ranged from 30–107 beds (mean 70 beds) at the commencement of the study. There were no for-profit facilities in the municipality, and private non-profit facilities were excluded from the trial as they had less affiliation to the municipal nursing home provider.

### Intervention group

The intervention was formed as a KT capacity program consisting of an educational component and a facilitation-upon implementation component.

### The clinical innovation

The decision to choose NEWS2 as the clinical innovation was undertaken in the IKT partnership. Practice Development Nurses (PDNs) hold a particular responsibility for KT and professional development in the organization and were considered key stakeholders when identifying a clinical area of collective relevance for the KT project [28]. Researchers organized a 60-min workshop, in an already scheduled meeting of PDNs. To prepare for the workshop, each PDN was asked to collect ‘clinical uncertainties’ in their respective nursing homes, as care staff had experienced over the last month. In the workshop, one common list of uncertainties was developed, then ranked according to the number of times the same area of uncertainty appeared. Researchers performed literature searches to determine which of them had sufficient level of evidence to pursue as a knowledge-gap. The highest ranked clinical uncertainty was how to assess clinical deterioration in the residents, using NEWS2. This priority was supported by nursing home physicians and the top management of the organization.

Although NEWS2 is a validated tool within acute care settings, the working group behind NEWS2, the UK Royal College of Physicians, suggest that it be adapted for use outside the hospital setting [29]. These adaptations should consider factors such as clinical competencies and patient socio-demographics. In our study, the necessary adaptations were made during a workshop that included the Head of physicians in the nursing home organization and his team. This process was thoroughly documented, and for transparency, the documentation has been translated and included in Additional file 1.

### Implementation strategy

The KT capacity program consisted of two distinct parts. Part I was an educational KT capacity program tailored to the KT needs of care staff [28] and lasting for the academic spring semester in 2019. During the program, participants from each facility developed local knowledge-to-action (K2A) plans for the implementation of NEWS2. Part II was a facilitation-upon-implementation period, where the PDN in each NH applied their KT competence, in locally developed K2A plans. Part II commenced when Part I ended, from June 2019 until March 31, 2020. More details about the intervention is available in Table 1, and in the GREET checklist (Additional file 2).

**Table 1:** Description of the educational component of the IMPAKT intervention

Name	The IMPAKT knowledge translation capacity program. A tailored, adaptive and multifaceted knowledge translation (KT) program	
	Part I: Educational KT capacity program*	**Part II**: Facilitation-upon implementation
***Actors***	Academic staff from Center for evidence-based practice (EBP) set up and delivered an educational KT capacity program. Learners/students were employed in intervention group NHs.	Facilitation led by members of the academic partner to Practice development nurses who led the implementation of a clinical innovation in their respective nursing homes. Facilitation was tailored to the challenges and needs that arised during an 8-month long implementation period.
***Actions***	To provide the NH organization with a KT capacity program tailored and adapted to the roles and needs in the organization. To increase readiness to deal with KT by equipping learners with competencies and tools in EBP and KT.	To facilitate a real-world KT project aiming to implement NEWS2, using the knowledge-to-action model to guide the process. Some facilitation activities were collective across the intervention NHs, including four meetings with PDNs in intervention group, to share their challenges and solutions over a common KT project. Others were specific to each NH, such as how they could achieve leadership involvement, and took place every two weeks on telephone.
***Target(s) of the action***	To enhance Practice Development Nurses’ (PDNs) with knowledge, skills and tools to lead KT-projects and professional development in the NH organization. To enhance knowledge, skills and attitudes related to partaking in KT-projects in NHs.	To support newly trained PDNs in their pursuit of a KT project in the NH. Help them use the tools and frameworks provided in the educational program, and keep on track with their local Action plans for the implementation of NEWS2 in their respective NHs.
***Temporality***	The capacity program was the initial activity in the intervention. Preceding the intervention was a development phase of the intervention, in an IKT partnership.	The facilitation period represented a continuation of part I. This period marks the implementation period the KT project and a shift from educational to clinical setting.
***Dose***	The program was delivered as a 15 ECTS university led EBP/KT program, with in-house sessions over one semester in university premises. The program had three distinct modules, reflecting three advancing steps of KT competence. The modules consisted of 2 + 3 + 2 full days, with some ‘homework’ between sessions in each NH. All modules were mandatory for PDNs, but each facility was encouraged to send more staff to the program.	Educational meetings over two days in a train-the trainer fashion to develop clinical capacity in the use of NEWS2. Each NH had three members of staff on a course with clinical staff from the local emergency room. Four physical implementation team meetings among the PDNs, over the course of 8 months. Bi-weekly telephone facilitation meetings between PDN and facilitator from academic partner.
***Implemen-tation outcomes affected***	Acceptability, measured as the number of participants throughout the program modules, and work in-between modules at each nursing home. Feasibility of program, using open-ended survey at end of each module and counting participants. Adoption of concepts of knowledge-to-action when assessing each NH action plans for implementing NEWS2.	Appropriateness of educational material developed to guide the PDNs in their work to plan, tailor, and carry out a KT project according to the steps of knowledge-to-action plan. Adoption of KT capacity, assessed by the PDNs’ ability to plan a knowledge-to-action process and then carry it out. Uptake of NEWS2 in nursing homes, assessed as calculated penetration of its use.
***Justification***	Within the IKT partnership we undertook a development period where we collected input from the range of staff in the NH organization towards the current level of EBP and KT competencies. Research was set up in an IKT partnership to enhance involvement and relevance [13, 30, 31]. Educational program underpinned by research [32] and long-standing experience in the academic team [33–35].	Many studies show that EBP courses change learners’ knowledge and attitudes positively, but that they struggle to change their behavior and apply EBP in clinical practice. This coincides with the academic partner’s experience [33, 34]. The facilitation period was undertaken to integrate and facilitate the transfer of learning from the educational program to the learners’ clinical setting.

*Further details about the educational program, see Additional file 2

### Control group

The control group continued with care as usual, without any interference from the IMPAKT project. Yet, several national and local initiatives overlapped with our clinical innovation that encouraged the use of NEWS2 in community settings. For instance, the Norwegian patient safety program published a national resource called “Early detection and fast response in somatic health deterioration”, where the NEWS2 tool was available online for all care settings in Norway [22]. At least two facilities in the control group participated in a local learning network, established to facilitate the use of NEWS2. In line with the pragmatic design of our trial, we made no attempt to mitigate other initiatives that could influence the adoption of NEWS in the control group.

### Outcomes

We assessed the effect of the implementation strategy based on the rate of documented use of NEWS2 at the resident level. Secondary outcomes included the use of NEWS2 in clinical situations with clear indications for NEWS2 assessment, defined as when residents acquired infections or were transferred to acute care.

### Sample size

A statistician calculated the a priori sample size based on an expectation of 10% improvement in the use of NEWS2 in the intervention group compared to the control group. To have a power of 80% to detect this difference between groups at a 5% level of significance, accounting for correlation between outcomes within the same clusters, we calculated that we needed minimum 470 residents or 7 nursing homes per arm. For more information about power calculations see Additional file 3.

### Enrollment and randomization

The nursing home organization committed to participation in the overall IMPAKT study funding was secured. Two facilities were considered unfit to participate by the Director of the NH organization and were excluded prior to randomization.

This study is a cluster randomized RCT. The rationale for cluster randomization is related to the practical challenge of randomizing the intervention across the nursing home population. Randomizing at the nursing home level, rather than using simple random sampling, is a more feasible approach often used in the healthcare setting.

Enrolled nursing homes were paired, based on a list of disidentified facilities, to match on size (number of beds) and type of beds (long-term beds or short-term beds). Subsequently, the NH pairs were randomly assigned 1:1 to either intervention or control using the random number generator in IBM SPSS statistics. This ensured concealment of the allocation. All residents residing in the nursing homes during the study period were included in this study.

### Data collection

Individual resident-level registry data was extracted from electronic patient journals. A Structured Query Language (SQL) syntax was developed and validated with the contractor of the electronic patient journal system (DIPS ASA), based on its codebook. The query was run by the IT department in the municipality.

### Blinding

Blinding was not possible among care providers, residents or researchers. Outcome assessors were blinded.

### Statistical methods

Firstly, a generalized additive model was used to smooth the outcome in presented graphs. Linear multilevel growth model analysis was used to assess the impact of the KT intervention on the use of NEWS2, which was measured by NEWS2 assessments per patient month. The statistical models accounted for the correlation between outcomes within the same cluster (NH id) and for repeated measurements among residents (resident id) over time. All analysis were done in the R statistical environment using the package “lme4” [36, 37].

The likelihood ratio test was used to examine best model fit for both fixed and random effects using a maximum likelihood estimator. For the fixed effects, likelihood ratio test was used to compare nested models to determine if the fixed effects being tested significantly improved the model fit. Specifically, we tested a three-way interaction effect (time*allocation*period) between the use of NEWS2 by time (per month), allocation to either intervention or control group, and period (baseline/clinical intervention/follow-up). In addition, we tested a four-way interaction to see if the secondary outcomes moderated the effect of the intervention on the use of NEWS2 (time*allocation*period*referral- or infection rates). The significance level was set at 0.05.

For the random effects, the higher order variance components were tested against a simpler model excluding higher-order interactions. In our study this meant assessing fit based on random intercept by resident id and nursing home id (a three-level model). A model with three-level random intercept and a fixed effect three-way interaction had the best model fit.

The intraclass correlation coefficient (ICC) was used to indicate the proportion of the total variance that could be explained by the group-level clustering. An ICC value greater than 0.05 or 5% is considered to indicate a meaningful variation at the group level. In this study the ICC value was 0.22 indicating a multilevel factor structure between nursing homes that should be accounted for.

The effect size measures will be expressed as the (unstandardized) mean difference (Md) and standardized mean difference (d). We calculated standardized mean difference for multilevel growth models (d_GMA-raw_) based on recommendations by Feingold [28]. This effect size can be interpreted similar to the Cohen’s d heuristic where an effect size of 0.2 can be considered a small effect, 0.5 represents a medium effect and 0.8 a large effect [38].

## Results

**Fig. 2:**
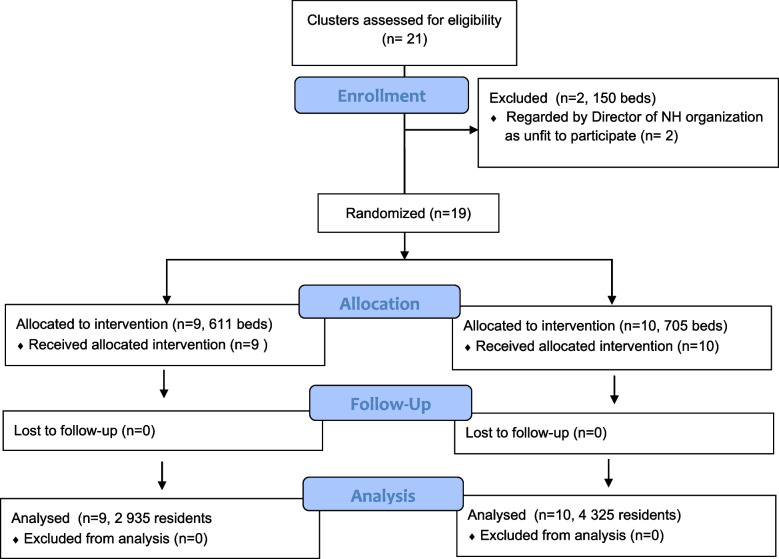
Participant flow and trial profile

Nineteen nursing home facilities were included and randomized to the intervention (*n* = 9) or control (*n* = 10) group. A total of 7 260 unique residents resided in the nursing homes during the study period. Participant flow and demographic characteristics of study population are shown in Fig. 2 and Table 2, respectively. Nursing homes in our sample were not significantly different based on size, referral rate to acute care and gender distribution, but we note some differences in the amount of long-term beds against short term beds (X^2^ = 34.36, df = 1, *p* < 0.001) and a slightly older sample (t = 6.26, df = 4946, *p* < 0.001) in the intervention group at baseline (Table 2). The nursing homes in this study were matched as closely as possible a priori so the small differences remaining can be attributed to real-world clinical practice differences and/or random chance.

**Table 2:** Demographic characteristics of intervention and control groups at baseline

Level	Variables	Intervention	Control
**Nursing home level**	Number of beds (mean)	611 (68)	705 (71)
	Number of long-term beds (%)	465 (76)	430 (60)
	Number of short-term beds (%)	146 (24)	275 (40)
**Resident level**	Age (SD)	84.4 (9.3)	82.9 (9.6)
	Female, %	62	60
	All-cause infection rate pr month, %	11.8	10
	Referral rate to acute care pr month, %	16.2	12.8

### Intervention effect on the use of NEWS2

**Fig. 3:**
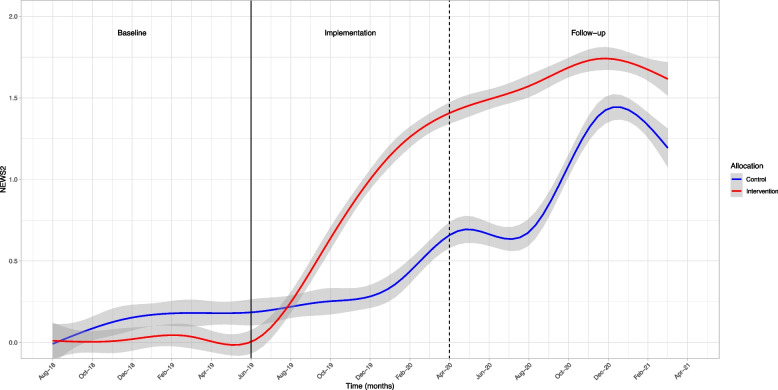
Documented NEWS2 assessments per patient month

Figure 3 shows the average amount of NEWS2 assessments by month in intervention and control groups. During the baseline period, the educational component of the intervention took place and NH stakeholders decided on a relevant clinical intervention. As shown in Fig. 3 and Table 3 NEWS2 was rarely documented used during the baseline period in either the intervention or control group.

**Table 3:** Growth model estimates of NEWS2 assessment per patient month and effect sizes

Allocation	Intervention period			Follow-up/COVID-19	
	August 2018	April 2020	Effect	March 2021	Effect
	Mean (95%CI)	Mean (95%CI)	Md (d)	Mean (95%CI)	Md (d)
Control	0 (-0.19, 0.19)	0.38 (0.19, 0.57)		1.46 (1.27, 1.65)	
Intervention	0 (-0.21, 0.21)	1.44 (1.23, 1.64)	1.06 (2.03)	1.75 (1.55, 1.96)	-0.77 (0.61)

*Md*  (unstandardized) mean difference, *d*  standardized mean difference

During the facilitation-upon-implementation period there was a substantial increase in NEWS2 assessments (Fig. 3 and Table 3). Based on the multilevel growth model analysis estimates, NEWS2 assessments increased from 0 to 1.44 (95% CI: 1.23, 1.64) per patient month in the intervention group and 0.38 (95% CI: 0.19, 0.57) in the control group. This represents a statistically significant and large mean difference of 1.06 NEWS2 assessments per patient month. In the last month of this phase, the use of NEWS2 was almost 4 times higher in the intervention group, compared to the control group (d = 2.03) (Table 3).

The use of NEWS2 continued to increase during the follow-up period, when the intervention period had ended (Fig. 3). Readers should note that the COVID-19 lockdown started in March 2020, and continued for the entire follow-up period. Results from the growth model analysis show that NEWS2 assessments continued to increase significantly to 1.75 (95% CI: 1.55, 1.96) assessments per patient month in the intervention group, in the final month of the follow-up phase. Somewhat unexpectedly, that largest increase during the follow-up period was found in the control group (Md = -0.77) which increased to 1.46 (95% CI: 1.27, 1.65) assessments per resident in the final month (d = 0.61) (Table 3).

### Secondary outcomes

All-cause infections and referrals to acute care were clinical conditions defined as indications for assessing NEWS2 by nursing homes staff. Both referrals and infections moderated the impact of the intervention on NEWS2 usage, based on the growth model estimates. In the baseline period, there was no statistical difference in NEWS2 usage when residents were referred to acute care or had an infection, in either of the groups. This changed during the implementation period. In the last month of the intervention period, residents in the intervention group were significantly more likely to have a NEWS2 assessment when acquiring an infection or being referred to an acute care facility. Compared to the control group, residents referred to acute care received 2.05 times more NEWS2 assessments, while residents with an infection received 1.89 more NEWS2 assessments in the intervention group. During the follow-up period, which coincided with the COVID-19 pandemic, the pattern shifted once more. Both the intervention and control groups staff continued to document the use of NEWS2 significantly more frequent for referrals and for residents with infectious diseases. Notably, the control group began using NEWS2 more often among referrals to acute care, with an increase of 0.68 measurements per resident and conducted 1.78 more assessments for infections in the last month of the follow-up, compared to the intervention group.

## Discussion

In summary, we evaluated a KT capacity program and its effectiveness on the implementation of the clinical tool NEWS2 in a nursing home organization. Compared to baseline use of the tool, as well as comparing the intervention and control groups, we found a large and significant effect. By the end of an extensive intervention period, each resident in the intervention group had on average 1.4 assessments per month. This was approximately four times more often than in the control group, representing an increase from close to zero use of the tool. Furthermore, the intervention group used the tool more appropriately, such as in cases of infections and referrals to acute care, both of which are clinical indications and prompts for assessing with NEWS2.

Our intervention period was scheduled to end March 30th in 2020, but as the COVID-19 erupted a few weeks earlier, our follow-up period coincided almost linearly to the outbreak. In this period, the use of NEWS2 not only sustained, but continued to increase in both the intervention and control groups. The pandemic called for intensified observations to identify cases early and monitor their progress. In the state of emergency, most resources were channeled to the handling of the outbreak, including extraordinary imposes from the government to keep the outbreak as low as possible. Despite concerted efforts that most certainly intensified the use of the tool in both groups, the quantity and quality of NEWS2 assessments remained higher for residents in the intervention group. Although we did not investigate patient outcomes such as mortality, we assume the large use of the tool reflects both relevance and usefulness in terms of support to clinical observations, decision making and communication.

The intervention was developed during a long-lasting IKT partnership where both sides of the collaboration gained insights to each other’s perspectives on the challenge at hand [26]. During this phase, KT roles and responsibilities in the organization was mapped, together with KT needs, that in turn informed the KT capacity program [28]. Inherently in the partnership was a significant investment on both sides of the collaboration to gain insight to each other’s perspectives of the problem, and strong management commitment. This ‘capital’ may very well have mediated the effectiveness of the capacity program itself, which is a known challenge in the assessment of impact to IKT research [39]. The IKT partnership was also important for adapting the NEWS2 instrument to the Norwegian nursing home context while maintaining its integrity and utility. Involving key stakeholders, such as the Head of Physicians, ensured that the clinical tool was aligned with local competency and patient needs.

The scarcity of both quantity and quality of KT research from the nursing homes setting has been demonstrated time and time again [4, 9]. Earlier studies do suggest that multifaceted implementation strategies are likely superior to single component strategies in this setting [9, 17, 18]. Suggestive from this research, is that addressing organizational complexity, can increase implementations success [13, 14]. In our IKT partnership, decision makers and researchers collaborated over a period of 18 months to map KT facilitators and barriers to develop the intervention [26]. We believe that our intervention success is largely a result of the IKT approach, so that implementation strategy matches the needs and determinants of the context of where it was deployed.

In context of what was already known about the effectiveness of tailored and multifaceted implementation strategies, our results demonstrated a larger effect size than observed in systematic reviews assessing similar strategies [9, 40, 41], where effect sized generally vary from small to moderate.

A commonality across nursing homes implementation studies is the lack of methodological robustness [4, 9, 17, 42]. For instance, many studies lack control groups, which makes authors of systematic reviews unable to draw conclusions about effect. Moreover, the lack of using recommended reporting taxonomies, make them challenging to compare and replicate.

An interesting overview of systematic reviews, with a theory-led analysis of what implementation strategies work and why, conclude that interventions based on education combined with a collective action tend to be more successful, than education alone [43]. The education component of our implementation strategy was central and tailored to the needs of care staff in nursing homes. Unlike many previous studies our intervention aimed to increase the general KT capacity, which we later facilitated during the implementation of NEWS2. Our educational program was well received by its main target group, and PDNs reported a shift to a more professional and structured mode of KT [44]. Moreover, they expressed a marked change towards a more organizationally vested KT approach, where stakeholders were more involved and held accountable during the implementation of NEWS2.

### Strengths and limitations

Our findings need to be considered in context of several strengths and limitations. Methodological strengths include a robust pragmatic RCT design, an extensive observation period and objectively measured patient level outcome data. The use of recommended taxonomies and checklists to describe the intervention in detail likely enhances the transparency and judgment of transferability.

In IKT projects, partners are expected to participate in the entire research process, including the interpretation of results. Due to COVID-19, planned activities such as interpreting the findings of the trial with stakeholders, were mostly undertaken by researchers. This has certainly led to missed opportunities in terms of insights to the intervention itself versus the IKT partnership. For the same reason, planned seminars to address sustainability of KT capacity were cancelled, and valuable insights towards the long-term value and sustainability of KT capacity was lost.

Finally, a cost–benefit analysis could have provided additional insight to the efforts and resources it takes to undertake an IKT partnership.

## Conclusion

The integration of KT to complex healthcare settings requires a multilayered approach. Our KT capacity intervention demonstrated a large effect on the use of NEWS2 among care staff in a large nursing home organization. The strategy was designed iteratively in an IKT partnership, where significant groundwork was laid down by both clinical and academic sides of the collaboration. The key ingredients to our intervention were simple with capacity building and facilitation, but well-founded in the context and with high levels of two-way commitment in the IKT partnership. Although resource-intensive, our intervention adds evidence to the fact that IKT strategies show promise in the delivery of effective implementation of evidence-based practices in complex healthcare settings, like nursing homes. The sustainability level of the KT capacity, though, remains unexplored.

## Supplementary Information

Supplementary Material 1.

Supplementary Material 2.

Supplementary Material 3.

Supplementary Material 4.

Supplementary Material 5.

## Data Availability

Restrictions apply to the availability of data. All data was extracted from the Electronic Patient Journal system. Since this study was exempted from patient consent the data were only made available for the purpose of this study and is not publicly available.

## Abbreviations

NEWS2: National Early Warning Score 2
LTC: Long-Term Care
KT: Knowledge Translation
IKT: Integrated Knowledge Translation
RCT: Randomized Controlled Trial
PDNs: Practice Development Nurses
NH: Nursing Homes
EWS: Early Warning System
SQL: Structured Query Language
ICC: Intraclass Correlation Coefficient
