# Supplementary material for: The effectiveness of a knowledge translation intervention on the implementation of NEWS2 in nursing homes, a pragmatic cluster RCT
Source: Implement Sci. 2024 Sep 11;19:64. doi: 10.1186/s13012-024-01392-6 (PMC11391697; doi:10.1186/s13012-024-01392-6)
Supplement: Supplementary file 1 — Supplementary Material 1. [file 13012_2024_1392_MOESM1_ESM.pdf]

Addition file 1. National Early Warning Score 2 (NEWS2) scoring.

Based on the NEWS2 scoring manual from Royal College of Physicians (2017).

The “clinical response” (page 2) was adapted to the Norwegian nursing home context. The adapted version is translated to English for the purpose of international readers.

NEWS 2 scoring system

Physiological parameter	Score 3	2	1	0	1	2	3
Respiration rate (per minute)	≤8		9–11	12–20		21–24	≥25
SpO_2 Scale 1 (%)	≤91	92–93	94–95	≥96			
SpO_2 Scale 2 (%)	≤83	84–85	86–87	88–92<br>≥93 on air	93–94 on oxygen	95–96 on oxygen	≥97 on oxygen
Air or oxygen?		Oxygen		Air			
Systolic blood pressure (mmHg)	≤90	91–100	101–110	111–219			≥220
Pulse (per minute)	≤40		41–50	51–90	91–110	111–130	≥131
Consciousness				Alert			CVPU
Temperature (°C)	≤35.0		35.1–36.0	36.1–38.0	38.1–39.0	≥39.1	

Note: If infection is suspected and NEWS2 is ≥ 5, immediately assess whether the patient might have sepsis and, if so, initiate sepsis treatment.

SpO2 Scale 2: Should only be used for patients with known hypercapnic respiratory failure with a goal of SpO2 between 88-92%, verified by blood gas analysis. The doctor should document in the record when Scale 2 should be used. In all other cases, Scale 1 should be used.

Level of Consciousness:

- A = Alert
- C = New confusion
- V = Voice (responds to voice)
- P = Pain (responds to pain stimulus)

- U = Unresponsive (does not respond to voice or pain stimulus)

Clinical response after score

NEWS SCORE	MONITORING FREQUENCY	CLINICAL RESPONSE	RISK OF HOSPITAL MORTALITY
0	Minimum every 12 hours	If the measurement is done based on clinical indication, it should be repeated at least once, as recommended under "monitoring frequency". If the measurement is a baseline measurement, no repeat measurement is needed until clinical indication arises.	Low
Total 1–4	Minimum every 4-6 hours	Note: The measurement must always be considered in conjunction with the patient's last baseline measurement. Does this represent a deterioration from baseline? Inform the responsible nurse, who must assess the patient. The responsible nurse decides on monitoring frequency and/or if clinical actions are required.	Low
Score 3 in one parameter	At least once per hour	Note: The measurement must always be considered in conjunction with the patient's last baseline measurement. Does this represent a deterioration from baseline? The responsible nurse contacts the responsible doctor, who assesses and decides on the monitoring frequency and whether further treatment measures are needed.	Low-medium
Total 5 or higher (Threshold for rapid response)	Minimum once per hour	Note: The measurement must always be considered in conjunction with the patient's last baseline measurement. Does this represent a deterioration from baseline? The responsible nurse contacts the responsible doctor. The responsible doctor decides on monitoring frequency and treatment level. The responsible nurse calls for additional help from medical personnel if the treatment level needs to be changed.	Medium

Total 7 or higher (Immediate response)	Continuous monitoring of vital functions	Note: The measurement must always be considered in conjunction with the patient's last baseline measurement. Does this represent a deterioration from baseline? The responsible nurse should immediately contact the responsible doctor or other emergency medical service. The responsible nurse ensures someone secures the patient's airway. Consider transfer to a higher level of care. Obtain information on decisions from the Decision Form in GBD. Further treatment at the appropriate level of care with defined intervals for monitoring is decided by the doctor.	High

Note. A low score does not rule out serious illness. NEWS2 is a supplementary tool for assessing vital functions in adult patients and must always be used in combination with the healthcare personnel's expertise and clinical judgment.

Reference

Royal College of Physicians. *National Early Warning Score (NEWS) 2: Standardising the assessment of acute-illness severity in the NHS.* Updated report of a working party. London: RCP, 2017.
