# Supplementary material for: The effectiveness of a knowledge translation intervention on the implementation of NEWS2 in nursing homes, a pragmatic cluster RCT
Source: Implement Sci. 2024 Sep 11;19:64. doi: 10.1186/s13012-024-01392-6 (PMC11391697; doi:10.1186/s13012-024-01392-6)
Supplement: Supplementary file 2 — Supplementary Material 2. [file 13012_2024_1392_MOESM2_ESM.pdf]

Additional file 2. GREET 2015 checklist for the educational component of the IMPAKT intervention.

BRIEF NAME

1. INTERVENTION: Provide a brief description of the educational intervention for all groups involved [e.g. control and comparator(s)].

The overall aim of the IMPAKT study was to improve KT capacity in a NH organization. The IMPAKT trial is a cluster randomized RCT evaluating the effectiveness of a tailored, adaptive and multifaceted knowledge translation (KT) capacity program on the implementation of the National Early Warning Score-2 (NEWS2). The capacity program held two components, where the first (part I) was an educational component and the second an implementation-upon facilitation component where key participants from the educational intervention were facilitated during a KT project in clinical practice, the implementation of NEWS2.

The educational component (part I) is described in this checklist.

In line with Straus's "Doers, users and replicators of EBP" (1, 2), we defined learners at different levels among the care staff in the NH organization, to reflect their role and responsibilities in terms of EBP/KT. The program was developed for different levels of learners and were reflected in the program's three modules. The first module is an introduction to EBP/KT and thus relevant to all care staff, or what Straus refers to as *replicators*. The second and third module is relevant to *users* of EBP and the latter was tailored to leaders of change in the NH organization. The control group did not receive any of the intervention components.

WHY - this educational process

2. THEORY: Describe the educational theories.

Education in EBP and KT is most often placed in the socio-constructivist paradigm. Several theories within this paradigm has direct influence to the teaching methods and pedagogy overall. Constructivism is a learning theory that emphasizes the active role of learners in constructing their understanding and knowledge. Rather than passively receiving information, the learners reflect on their experience and incorporate new knowledge within their own apprehension, which promotes a deeper learning and understanding (3).

Bandura's social learning theory emphasizes the role of social interactions and observational learning in the shaping of behavior. In EBP education the impact of peer learning and collaborative learning is considered important, and for the same reason small group work is key throughout the program (4). *Self-efficacy* is a key term that stems from social learning theory and refers to that how people deal with situations is based upon how the same people judge their own ability to deal with them. By enhancing the learners' KT competence, we attempted to empower them to actively deal with KT in their real-world NH setting.

We utilized that learners were all from one NH organization, and members of a *community of practice* (COP) (5). COP may be viewed as a tool to enhance knowledge and improve practice, beyond personal learning, to improve organizational performance. Learners are brought together to think, learn and problem-solve together, on the relevance of EBP and KT.

Teachers on the program are more facilitators of learning than educators towards this community, and actively involved learners in populating the dynamic didactive relationship model (6) of the program in terms of aims, content, qualifications (or lack of), pedagogical facilitation and assessment.

As the target group consist of more and less experienced nursing staff in a NH organization, *adult learning theory* underpins the program. Representatives from the learners were involved in defining learning objectives (LOs), through focus group interviews, as part of the development of the intervention. The LOs were then presented in the program curriculum of study program. Then, at the first commencement of each of the three modules, the LOs were explicitly expressed and discussed, to ensure relevance for learners and embedded in the program. The program was organized so that learners should feel that they were in a position to influence the program and take control of the learning by adding context-specific relevance. Learning activities were linked to learners' everyday NH practice and set up to activate the learners. For instance, after a lecture on asking searchable and specific clinical questions, learners were applying their own clinical queries in frameworks such as PICO, and types of clinical questions (therapy, diagnosis, prognosis etc.).

CONCEPTS

Evidence-based practice (EBP) and knowledge translation (KT) were the two main concepts taught in the program. The research team had developed learning material over many years within EBP and more recently for KT, including digital and online resources and a workbook in Norwegian (7,8). Both digital and physical resources were actively used in the syllabus. For KT, the knowledge-to-action framework formed the basis for

APPROACH

Although teaching and learning approaches on the program were blended, all activities were planned to achieve student activity. The blend of approaches included classroom lectures, small group work, problem-based tasks. An online learning platform, or a learning management system (Canvas) was used to communicate with students, to provide students with curriculum and syllabus and to make available presentations.

3. LEARNING OBJECTIVES: Describe the learning objectives for <u>all</u> groups involved in the educational intervention.

The entire program was organized with Practice development nurses (PDNs) as the main target group, who has a main responsibility for KT and quality of care in the NH facilities. Typically, each NH facility as a PDN. But it was a wish from the NH organization that the program should be relevant to all care staff in the organization. Based on focus group interviews in the development phase of the intervention, we identified who should be defined as learners and what the learning objectives were to be. Practice development nurses (PDNs) were identified as having a key role in knowledge translation in the NHs and thus was our main target group for the intervention. Because learners had different roles and responsibilities in terms of KT and EBP in the organization, the program was designed as a staircase. Rather than to define different learning outcomes for different groups of learners, we defined LOs at the three different modules, where the first module was the most basic, and the two next more advanced. In the table below, the learning objectives and outcomes for the three different modules are outlined.

Table 1. Learning objectives and outcomes at the different modules of the program.

**Module and its aim**	**Knowledge**<br><br>*The learner…*	**kills**<br><br>*The learner…*	**General competence**<br><br>*The learner…*
Module 1:<br><br>To provide learners with basic competencies in evidence-based practice	Knows which sources of knowledge make up the definition of evidence-based practice.	Can identify areas of own clinical practice where there exist uncertainty in terms of what is 'best practice'.	Has gained insight to evidence-based practice and can apply this by contributing to change and quality improvement in the clinical setting.
	Knows that the process of evidence-based practice can be expressed in the model Steps of EBP.	Can reflect upon the significance of evidence-based practice in his or her own workplace.	
	Knows about tools and principles for each of the modules in EBP and where to find them.	Can explain to others what evidence-based practice is, its definition and its significance to clinical practice.	
	Knows the standing of evidence-based practice in society.	Can tell the difference of original research and systematically synthesized research.	
	Knows about the National electronic health library and how it is structured.	Can participate in the planning of implementing a change, using tools designed for the different modules of the knowledge-to-action model.	
	Is familiar with *The knowledge-to-action model* as a framework for implementing change.	Can navigate the National electronic health library to identify synthesized research evidence.	
Module 2:<br><br>To provide learners with competencies to distinguish evidence synthesis from original research; to find	Knows what characterizes a systematic review.	Can explain the characteristics of systematic reviews and clinical guidelines.	Has insight to the difference between original research and synthesized research and its significance to evidence-based practice.
		Can assess the quality of synthesized research, both systematic reviews and clinical guidelines.	
	Knows what characterizes evidence-based clinical guidelines.	Can reflect upon the relevance of systematically synthesized evidence as the foundation for change and knowledge translation in clinical practice. evidence-based practice in own clinical practice.	

evidence syntheses effectively; to critically appraise it, and to apply it in clinical practice.	Knows the role of synthesized research as the foundation for change in clinical practice.	Can effectively find systematic reviews and evidence-based clinical guidelines in the National electronic health library.	Can take active part in professional clinical discussions about the evidence-based practice in their own workplace.
	Knows scientific terms, relevant to evidence-based practice.		
	Knows sources for synthesized research.		
Module 3: To equip the learners with established terms, concepts and tools in knowledge-to-action to increase their readiness for real-world knowledge translation challenges.	Has breadth and depth insight about the knowledge-to-action model as a framework for knowledge translation in own work.	Can apply the generic knowledge -to-action model on a clinical problem.	Can plan, carry out and lead the implementation of evidence-based practice using the knowledge-to-action framework.
	Knows about different tools to assess practice in different modules of the knowledge-to-action process.		Can express and exchange viewpoints from their experience in applying the knowledge-to-action theory and tools during a real-world implementation endeavor.

4. EBP CONTENT: List the foundation modules of EBP (ask, acquire, appraise, apply, assess) included in the educational intervention.

Module 1of the program was tailored to care staff with no foreknowledge of EBP. This was for learners who perhaps had heard and seen the term but who were largely unfamiliar with key and basic principles of EBP. This unfamiliarity included what types of evidence to consider and the process of EBP (EBP steps). This step was designed to fit learners with no formal role and responsibility for EBP in their organization. Still, this group make up a large group in any healthcare organization and is considered important that they have a healthy critical attitude to their own and colleagues' practice, and to what evidence constitutes as evidence in EBP. During module 1, learners were introduced to the basics of evidence-based practice. What is it and

what significance does it have in our NH organization? Those were questions that learners had to dwell upon, answer and articulate. Learners were introduced to two models of EBP: the steps of EBP and the one who mirrors the definition of EBP, types of scientific and non-scientific evidence, and the importance of context and users.
Learners taught how to formulate specific (and searchable) questions based on clinical queries. Learners were introduced to The National Electronic Health Library as their first-hand resource for research evidence, as well as an online resource for EBP.

We leaned ourselves to the Sicily statement when developing this module and when we defined who the learners were in the organization (9). The statement says that "All health care professionals need to understand the principles of EBP, recognize EBP in action, implement evidence-based policies and have a critical attitude to their own practice and to evidence. Without these skills, professionals and organizations will find it difficult to provide 'best practice'".

In Module 2 of the program, our target group where people in any kind of leadership positions in the organization. Leaders in healthcare organizations in Norway are subject to a National statutory that regulates their responsibilities to ensure quality of care (10, 11). These are people in the organization which are expected to actively act upon and use research evidence in their job and thus will need to have ability to find the research and critically appraise it, or what we previously referred to as *user of EBP*. An important adjustment we made to the program, and which differs from earlier versions of the course, was to focus primarily on synthesized research evidence to inform changes in practice. In earlier versions, both original and synthesized research had their place in the program, including critical appraisal of different kinds of original research. This time, learners were taught the difference between original and synthesized evidence and the rationale for why SRs are the basic unit of research in EBP. In this part of the program, the focus was to acquire and appraise systematic reviews and guidelines.

Module 3 of the program was designed to give healthcare professionals with a particular responsibility for the implementation of EBP in the organization, the knowledge, skills and tools to work systematically with knowledge translation, or the application and assessment steps of EBP. We used the knowledge-to-action framework as the framework of choice, and used resources that underpinned this. For instance, we developed an Action plan template, to serve as a roadmap and guide to work systematically with implementation, including how to choose the relevant evidence-base for the clinical intervention, to involve relevant stakeholders to populate a good implementation strategy, to set aside sufficient time for the implementation project and to plan for assessing the work.

WHAT

5. MATERIALS: Describe the specific educational materials used in the educational intervention.
The learners were encouraged to use the **online learning platform** (Canvas), where a digital classroom was set up that gathered all resources and materials relevant for the course participants. Although the program ran over four months (one semester), they had face-to-face meetings 2+3+2 days, for modules 1,2 and 3 of the program, respectively. Most of the learners had been away from higher education for long and we made an effort to accommodate and remove their digital barriers in the classroom. Thus, we made it a priority to present in

simple manners, with deeper levels of information if interested. For instance, the digital classroom mirrored the curriculum in terms of the three modules, which was recognizable to learners. Instructions, learning outcomes and tasks were set up with detailed instructions for each of the modules of the program, and were presented and linked to materials in an easy-to-follow manner.

The face-to-face meetings were supported by **Norwegian learning resources**, including **textbook** and **electronic resources** in the National Health Library (7, 8).

For the apply and assess steps, we developed translated and adapted material based on existing international KT support. For instance, we first translated the **RNAO Toolkit: Implementation of Best Practice Guidelines**, then developed an **Action Plan template** as a short and adapted version.

At the level of "users" and "replicators" (2), learners were conceptually introduced to the steps of EBP, supported by Norwegian learning resources. Some Norwegian resources existed, others were adapted from international existing resources.

6. EDUCATIONAL STRATEGIES: Describe the teaching / learning strategies (e.g. tutorials, lectures, online modules) used in the educational intervention.

The different steps of The KT competence stairs entailed 2 + 3 + 2 face-to-face meetings in the university, for modules 1-3 respectively. We encouraged the learners to prepare in advance, by referring them to relevant resources in advance. The sessions were mainly tutorials, where student activity was encouraged and expected. In between the tutorials, participants had homework. We placed the responsibility for leading the homework

7. INCENTIVES: Describe any incentives or reimbursements provided to the learners.

No incentives were received, neither by the participants at the program, nor the NHs in the intervention group. It was a premise from the set-out to organize the educational program as genuine as possible, with no extra resources provided.

But the entire NH organization participated in the development of the intervention. And the top-management played a key role in the integrated KT partnership that underpinned the IMPAKT intervention. With input from all levels, they helped us identify main targets for KT and EBP, in addition to learning needs.

The main target group for the educational program were Practice development nurses. It was communicated from the top-management of the NH organization that they were expected to participate in the entire program (modules 1-3), if their NH was randomized to the intervention arm of the trial. Each NH director in intervention group was informed about the different modules of the educational program and were encouraged to give priority to send more participants, in addition to their PDN.

The NH organization received no compensation for the time the care staff spent in the educational program and had to find resources within their facility to replace clinical staff in shift roster. Staff who volunteered to participate were recruited based on motivation, and a decision within each facility about how many learners they sent.

WHO PROVIDED
8. INSTRUCTORS: For each instructor(s) involved in the educational intervention describe their professional discipline, teaching experience / expertise. Include any specific training related to the educational intervention provided for the instructor(s).<br>Module 1 – day 1 (see schedule)<br>K.A.: Associate professor, midwife, Master's degree in EBP, extensive experience in teaching introduction to EBP.<br>Module 1 – day 2<br>H.H.: Nurse from the regional Development center for NHs and home-based care (EBP, patient safety and quality improvement)<br>H.S.: Research librarian, Master's in EBP, extensive experience in teaching searching for research, as well as formulating precise and answerable questions.<br>M.W.N.: Professor and leader of community emergency and acute care health room.<br>B.G.: PI of the IMPAKT project and associate professor. Experienced in teaching and supervising on the Master's program in EBP (including KT).<br>R.E: Director of the nursing home organization in Bergen municipality.<br>Module 2 – day 1<br>B.E.: Professor in epidemiology, experienced in teaching at Master's program in EBP.<br>Module 2 – day 2<br>A.D.: Master's of EBP and advisor at the regional health trust. Experienced in both developing and teaching of clinical guidelines and EBP.<br>Module 2 – day 3<br>B.T.: Research librarian.<br>G.A.: Research librarian.<br>Module 3 – day 1+2<br>B.G.: PI of the IMPAKT project and associate professor. Experienced in teaching and supervising on the Master's program in EBP (including KT).
HOW
9. DELIVERY: Describe the modes of delivery (e.g. face-to-face, internet or independent study package) of the educational intervention. Include whether the intervention was provided individually or in a group and the ratio of learners to instructors.<br>All in-house sessions were provided face-to-face.
WHERE

10. ENVIRONMENT: Describe the relevant physical learning spaces (e.g. conference, university lecture theatre, hospital ward, community) where the teaching / learning occurred.

A university classroom was uses as a base for all inhouse sessions. For small group work, groups were encouraged to leave classroom and gather somewhere else, to solve the tasks.

WHEN and HOW MUCH

11. SCHEDULE: Describe the scheduling of the educational intervention including the number of sessions, their frequency, timing and duration.

See table 2 for a detailed timetable of the face-to-face education.

Table 2. Spring 2019 Course Schedule – Knowledge translation capacity program. Western Norway University of Applied Sciences (HVL)*

**Time**	**04.02.2019**	**05.02.2019**	**11.03.2019**	**12.03.2019**	**13.03.2019**	**25.03.2019**	**26.03.2019**
08:15-09:00	About the IMPAKT project (part I and part II) by B.G. and R.E.	Evidence-Based Practice Patient Safety and Quality Improvement by H.H.	Introduction to Systematic Reviews. How to Understand Statistics by B.E.	Introduction to Evidence-based Clinical Guidelines by A.D.	Search with a Librarian by B.T. and G.A.	Knowledge-to-Action Model by B.G.	Student presentations
09:15-10:00	Introduction to Evidence-Based Practice by K.A.	Study Design by H.S.	Introduction to Systematic Reviews. How to Understand Statistics by B.E.	Introduction to Evidence-based Clinical Guidelines by A.D.	Search with a Librarian by B.T. and G.A.	Knowledge-to-Action Model by B.G.	Student presentations
10:15-11:00	Introduction to Evidence-Based Practice by K.A.	Summarized Knowledge vs. Primary Studies. Knowledge Pyramid by H.S.	Introduction to Systematic Reviews. How to Understand Statistics by B.E.	Introduction to Evidence-based Clinical Guidelines by A.D.	Search with a Librarian by B.T. and G.A.	Action Plan by B.G.	Student presentations
11:00-11:45	Lunch	Lunch	Lunch	Lunch	Lunch	Lunch	Lunch
11:45-12:30	Introduction to Evidence-Based Practice by K.A.	Health Library Alerts – when new studies emerge by H.S.	Critical Appraisal of a Systematic Review by B.E.	Critical Appraisal of Guidelines by A.D.	Search with a Librarian by B.T. and G.A.	Action Plan by B.G.	Student presentations
12:45-13:30	Formulating Precise Questions; PICO by H.S.	What is Needed to Succeed with Evidence-Based Practice in an Organization by M.W.N.	Critical Appraisal of a Systematic Review by B.E.	Critical Appraisal of Guidelines by A.D.	Search with a Librarian by B.T. and G.A.	Action Plan by B.G.	Student presentations

13:45-14:30	Formulating Precise Questions; PICO by H.S.	Introduction to Implementation and Study Requirements by B.G.	Critical Appraisal of a Systematic Review by B.E.	Summary of the Day and the Way Forward (evaluation)	Search with a Librarian by B.T. and G.A.		Student presentations

*Mandatory activities

12. Describe the amount of time learners spent in face to face contact with instructors and any designated time spent in self-directed learning activities.

Face-to-face sessions amounted to 7 full days. Self-directed learning amounted to a minimum of 3 workdays. Whether the learner wanted to achieve academic credits for the program was voluntary and depended on the undertaking of a home exam.

PLANNED CHANGES

13. Did the educational intervention require specific adaptation for the learners? If yes, please describe the adaptations made for the learner(s) or group(s).

The educational intervention was developed based on the teaching team's previous experience running short courses in EBP. But in addition, efforts were made to get hold of local and contextual learning needs in the NH organization. We refer to this time period ahead of the actual intervention as the development phase of intervention, where we worked closely in an integrated knowledge translation partnership with the top management team of the NH organization. Based on several inquiries with different stakeholders, among other things to identify the learners and their needs, we made adaptations to the original short course program. These changes were made to the curriculum ahead of the intervention. Two main changes were made to the program. Firstly, we specifically designed the program to "users" and "replicators" of EBP. This was based on our investigations in the development phase, where we gained insights to everyday NH practice, and specifically how the practice development nurses worked and fundamentally struggled in finding and assessing research. In this program we lend ourselves to systematic reviews as the basic unit of knowledge upon which to base clinical practice upon. This made us bypass original research and focus solely on systematic reviews. Much of the time, teaching resources and activities that was saved by this change, was placed in another major adaption we made to the program. This change included to place a major focus on applying evidence, using the knowledge-to-action model as the framework.

UNPLANNED CHANGES

14. Was the educational intervention modified **during** the course of the study? If yes, describe the changes (what, why, when, and how).

The educational intervention was not modified during the course, but adapted to the learners input and needs.

HOW WELL

15. ATTENDANCE: Describe the learner attendance, including how this was assessed and by whom. Describe any strategies that were used to facilitate attendance.

We registered all attendees every day, by signature from learners. Although attendance was not mandatory, it might have been understood as such by learners. In the recruitment process, it was stressed that only learners who were motivated should sign up. The fact that the educational program was part of the integrated knowledge translation project, where top-management of the organization were actively involved, might have had an effect on attendance.

We encouraged each NH-director to recruit more learners than their professional development nurse. They could send as many as they wanted, but to attend module 2, you needed to have attended module 1. We signalled clearly, that the PDN had little choice but attending all three modules. Below is a table showing attendance for each of the modules of the program.

Table 3. Attendance from each NH facility in each of the modules of the program.

**Intervention group cluster (nursing home)**	**Participants**		
	**Module 1**	**Module 2**	**Module 3**
**1**	**4**	**1**	**1**
**2**	**2**	**2**	**2**
**3**	**4**	**4**	**2**
**4**	**5**	**3**	**0**
**5**	**7**	**7**	**7**
**6**	**4**	**3**	**3**
**7**	**11**	**7**	**7**
**8**	**5**	**5**	**5**

9	11	4	3
N=9 intervention clusters	Participants on module 1 (n= 53)	Participants on module 2 (n=26)	Participants on module 3 (n= 30)

16. Describe any processes used to determine whether the materials (item 5) and the educational strategies (item 6) used in the educational intervention were delivered as originally planned**.**

The materials (item 5) and educational strategies (item 6) were used and delivered as originally planned. The learning material is documented in the digital learning platform, and can be compared to the schedule set a-priori.

The program description for the educational intervention was developed in line with the University's policies for development of formal curricula. The course was approved within our university and is equivalent to 15 European Credit and Accumulation Transfer System (ECTS). The time schedule was set apriori and made available to the learners in advance. The schedule provides information about topics covered in the sessions.

17. Describe the extent to which the number of sessions, their frequency, timing and duration for the educational intervention was delivered as scheduled (item 11).

The number of sessions were delivered as set out in the time schedule for the course.

***based on the TIDieR guidance. We strongly recommend reading this statement in conjunction with the GREET 2015 explanation and elaboration paper for important clarifications on all the items. If relevant, we also recommend reading the TIDieR guidance (Hoffman et al. 2014)**

References

1. Straus SE, Green ML, Bell DS, Badgett R, Davis D, Gerrity M, et al. Evaluating the teaching of evidence based medicine: conceptual framework. Bmj. 2004;329(7473):1029-32.
2. Straus SE, Glasziou P, Richardson WS, Haynes RB. Evidence-based medicine E-book: How to practice and teach EBM: Elsevier Health Sciences; 2018.
3. Vygotsky, Lev Semenovich, and Michael Cole. *Mind in society: Development of higher psychological processes*. Harvard university press, 1978.
4. Bandura, Albert, and Richard H. Walters. *Social learning theory*. Vol. 1. Englewood Cliffs, NJ: Prentice hall, 1977.
5. Wenger E. Communities of Practice: Learning, Meaning, and Identity. Cambridge: Cambridge University Press; 1998.

6. Hiim H, Hippe E. Praksisveiledning i lærerutdanningen: en didaktisk veiledningsstrategi. Oslo: Gyldendal akademisk; 2006.
7. Nortvedt MW, Jamtvedt G, Graverholt B, Wøhlk Gundersen M. Jobb kunnskapsbasert!: en arbeidsbok: Gyldendal; 2021.
8. Helsebiblioteket.no [Internet]. Kunnskapsbasert praksis. Available from: https://www.helsebiblioteket.no/innhold/artikler/kunnskapsbasert-praksis/kunnskapsbasertpraksis.no
9. Dawes M, Summerskill W, Glasziou P, Cartabellotta A, Martin J, Hopayian K, et al. Sicily statement on evidence-based practice. BMC medical education. 2005;5(1):1-7.
10. Forskrift om ledelse og kvalitetsforbedring i helse- og omsorgstjenesten. Forskrift om ledelse og kvalitetsforbedring i helse- og omsorgstjenesten. 2017.
11. Helsedirektoratet. Veileder til forskrift om ledelse og kvalitetsforbedring i helse- og omsorgstjenesten. 2017.
