# Supplementary material for: The effectiveness of a knowledge translation intervention on the implementation of NEWS2 in nursing homes, a pragmatic cluster RCT
Source: Implement Sci. 2024 Sep 11;19:64. doi: 10.1186/s13012-024-01392-6 (PMC11391697; doi:10.1186/s13012-024-01392-6)
Supplement: Supplementary file 3 — Supplementary Material 3. [file 13012_2024_1392_MOESM3_ESM.pdf]

Additional file 3. Sample size calculation.

Step 1: Estimating sample size without taking account of clustering (individual randomization)
Parameters:
Number of nursing homes (clusters) with information on number of beds (size) = 22
Nursing home size range = 20 to 129
Mean nursing home size (m) = 72
Standard deviation of nursing home size = 31.21
Baseline proportion of outcome (NEWS2) in control arm (μ_1) = 0
Expected Proportion in intervention arm (μ_2) = 0.10
Target difference (μ_1- μ_2) = 0.10
Assumed intra-cluster correlation coefficient (ICC; ρ) = 0.05
Significance level (α) = 0.05
Power level (β) = 80%

Formula:
Variance of outcome "σ^2" = $\mu_1 (1- \mu_1) + \mu_2 (1- \mu_2)$
Sample size under individual randomization:

$$N = \frac{2(Z^{\alpha}/_{2} + Z_{\beta})^2(\sigma^2)}{(\mu_1 - \mu_2)^2}$$

Assuming that baseline rate in the reference population is 0%, and after applying continuity correction, the study would require a sample size of: ***90 for each group*** (i.e. a total sample size of 180, assuming equal group sizes), to achieve a power of 80% for detecting a difference in proportions of 0.10 between the two groups (test - reference group) at a two sided p-value of 0.05. In other words, if you select a random sample of 90 from each arm, and determine that 0% and 10% of subjects in the two groups have the outcome of interest, you would have 80% power to declare that the two groups have significantly different proportions at a 5% level of significance.

Step 2: Estimating sample size taking account of clustering but ignoring variation in cluster size
Using estimated sample size to calculate nursing home as the unit of randomization. Accounting for a correlation in the outcomes within the same nursing homes.

Inflation factor = 1 + (m-1) ρ = 4.55

Sample size taking account of clustering = 90 x 4.55 = 410

Number of nursing homes needed per arm = 410 / 72 = 6

Step 3: Estimating sample size taking account of clustering and variation in cluster size
Accounting for both correlation in the outcomes within the same nursing home and nursing home size difference.

Cluster variation coefficient "CV" = *Standard deviation of cluster size* / *Mean cluster size = 0.435*

Inflation factor = 1 + (m (1 + CV^2) -1) ρ = 5.22

Sample size taking account of clustering & variation in cluster size = 90 x 5.22 = 470

Number of nursing homes needed per arm = 470 / 72 = **7 per am**
