# Supplementary material for: The effectiveness of a knowledge translation intervention on the implementation of NEWS2 in nursing homes, a pragmatic cluster RCT
Source: Implement Sci. 2024 Sep 11;19:64. doi: 10.1186/s13012-024-01392-6 (PMC11391697; doi:10.1186/s13012-024-01392-6)
Supplement: Supplementary file 4 — Supplementary Material 4. [file 13012_2024_1392_MOESM4_ESM.pdf]

REK
REGIONALE KOMITEER FOR MEDISINSK OG HELSEFAGLIG FORSKNINGSETIKK

The Project Birgitte Graverholt

Regional Committee for Medical & Health Research Ethics
South East Norway, Section C
Postbox 1130 Blindern
NO-0318 Oslo
Norway

Phone: + 47 22 84 55 98
E-mail: t.t.mosling@medisin.uio.no
Webportal: http://helseforskning.etikkom.no

Our ref.: 2019/1255C

Date: 30st of November 2021

To whom it may concern,

Re: REC Letter of Confirmation

I am writing in reference to a request from Birgitte Graverholt, regarding a Letter of Confirmation in English.

Confirmation
We hereby confirm that Regional Committee for Medical & Health Research Ethics, Section C, South East Norway, approved the Research Project: «Implementation of Early Warning Scores (EWS) for early detection of deterioration among nursing home residents». (Norwegian title: Implementering av verktøy for tidlig oppdagelse av forverret tilstand i sykehjem) at its Committee Review Meeting on the 22th of August 2019.
The Project Manager for the study is Birgitte Graverholt, and the Institution Responsible for Research is Høgskulen på Vestlandet.

The approval has been given on the basis that Research Project will be implemented as described in the Research Protocol.

Ethics Committee System
The Ethics Committee System in Norway consists of seven Independent Regional Committees with authority to either approve or disapprove Medical Research Studies conducted within Norway, or by Norwegian Institutions, in accordance with the Act on Medical and Health Research (2008).

Please do not hesitate to contact the Regional Committee for Medical and Health Research Ethics Section South East C (REK Sør-Øst C) if you have any questions, as we are happy to help.

Yours faithfully,

Erik Fosse
Chair of the Regional Committee for Medical
& Health Research Ethics of South East Norway,
Section C

Tone Transeth Mosling
Executive Officer
